# Comprehensive Evaluation of Inflammatory Biomarkers and Osmolarity to Distinguish Simple and Complex Febrile Seizures in Children

**DOI:** 10.3390/children10101594

**Published:** 2023-09-24

**Authors:** Özlem Erdede, Erdal Sarı, Emek Uyur, Rabia Gönül Sezer Yamanel

**Affiliations:** 1 Department of Pediatrics, Zeynep Kamil Maternity and Children’s Disease Training and Research Hospital, University of Health Sciences, 34668 Istanbul, Turkey; erdalsari@gmail.com (E.S.); rabiagonul@hotmail.com (R.G.S.Y.); 2 Department of Pediatric Neurology, Zeynep Kamil Maternity and Children’s Disease Training and Research Hospital, University of Health Sciences, 34668 Istanbul, Turkey; emekuyur@gmail.com

**Keywords:** biomarkers, osmolarity, complex febrile seizure, simple febrile seizure

## Abstract

With limited sample sizes and varying study outcomes regarding complete blood count (CBC)-associated biomarkers and their febrile seizure (FS) classification, along with limited research on osmolarity, this study aims to evaluate CBC-associated biomarkers, including osmolarity, for a comprehensive view of their diagnostic value. This single-center retrospective study used data from 364 children (aged 5–60 months) diagnosed with FS. The patients were categorized into simple FS (*n* = 221) and complex FS (*n* = 143) groups. CBC and biochemical tests, including sodium, potassium, chloride, glucose, blood urea nitrogen, and C-reactive protein levels, were evaluated. The neutrophil-to-lymphocyte ratio (NLR), mean platelet volume-to-lymphocyte ratio, and osmolarity were calculated and compared between FS types and the number of seizures. Receiver operating characteristic (ROC) curve analysis was conducted to assess the predictive utility of these markers. Inflammatory markers, including NLR, were ineffective in predicting FS types. Complex FS cases exhibited a significantly lower osmolarity than simple FS cases. The area under the ROC curve for osmolarity to distinguish complex FS was 0.754, while other markers did not reach the desired threshold of 0.700. Including osmolarity in the classification of FS has clinical applicability. Physicians may consider osmolarity as an additional tool to aid in clinical decision-making.

## 1. Introduction

Febrile seizures (FSs) are the most prevalent benign seizure type related to age among children globally. Although the incidence of FSs varies across studies, it generally affects approximately 2–11% of children, typically between the ages of 6 months and 5 years [1,2].

Abnormal brain responses to elevated temperatures lead to the occurrence of FSs [3]. In order to diagnose FSs, the body temperature should exceed 38 °C during a feverish illness. According to the American Academy of Pediatrics guidelines, a FS is characterized by a seizure occurring in conjunction with fever without a central nervous system infection. This applies to a child with no history of FSs, neurological disorders, or conditions that increase susceptibility to seizures [4,5]. The exact cause of FSs remains unclear and is believed to arise from multiple factors. Among the well-recognized risk factors are viral infections, certain vaccines, genetic susceptibility, and nervous system development under conditions of heightened stress [6,7]. A simple FS typically lasts for less than 15 min and does not recur within a 24 h period. The predominant seizure type is generalized clonic, although atonic and tonic seizures can also occur. According to its definition, while a simple FS can extend for up to 15 min, most such seizures are brief, with a median duration of 3–4 min [8]. Following a simple FS, children frequently and swiftly revert to their usual state. However, as with non-FSs, the postictal phase may entail confusion, restlessness, and drowsiness.

A complex FS encompasses seizures that originate focally, extend beyond 15 min, or recur within 24 h. This manifestation is prevalent, constituting approximately 20% of FS cases in most reported series. Prolonged seizures are observed in < 10% of cases, and focal seizures occurr in <5% of children experiencing FSs. Complex FSs can manifest after the initial occurrence of a simple FS. Nevertheless, the first seizure is frequently observed when examining children with complex FSs. Children with complex FSs are typically younger and tend to develop atypical seizures [8]. After the initial episode, recurrence is anticipated in approximately 30–50% of patients [9]. The occurrence of complex febrile seizures is linked to a heightened risk of developing temporal lobe epilepsy and other long-term cognitive effects, highlighting the need for an accurate diagnosis and effective management strategies [10,11]. Indeed, some studies provide evidence for the involvement of the interleukin (IL)-1, IL-6, and IL-8 cytokine systems concerning complex FS epilepticus in children. Circulating copeptin exhibits a noteworthy diagnostic accuracy in FSs and could serve as a valuable additional tool for the precise diagnosis of postictal states in emergency scenarios. Elevated plasma concentrations of IL-6 and adiponectin could potentially function as supplementary biomarkers for the early intervention or monitoring of children with FSs [12,13,14]. Cytokines exhibit rapid fluctuations in concentration, which makes their measurement more complex. Additionally, cytokines are present in very low concentrations in biological samples, which can require highly sensitive and specialized techniques for detection. A significant issue associated with these markers is their limited accessibility and elevated expenses.

Inflammatory indicators extracted from routine blood samples, such as the neutrophil-to-lymphocyte ratio (NLR), mean platelet volume (MPV), and MPV-to-platelet Ratio (MPR), could potentially be useful for differentiating between various types of FSs [15,16,17]. Research in this area has yielded inconsistent results regarding the diagnostic efficacy of biomarkers derived from routine blood parameters [18]. At present, research on the relationship between osmolarity and FSs is limited. Only a few studies have examined the direct connections between these two factors [18]. Osmolarity values, which reflect fluid balance in the body, could potentially affect neurological conditions and seizures. However, further comprehensive studies are required to enhance our understanding of these phenomena. Such studies may provide a clearer understanding of the association between osmolarity and FSs.

Despite the progress in identifying potential biomarkers, the existing literature reveals gaps hindering their seamless translation into clinical practice. Divergent findings concerning the diagnostic value of routine blood parameters and complexities of detecting cytokine levels in standard clinical assessments under specific healthcare conditions have led to uncertainties. Because of the varying outcomes in studies related to biomarkers and the inadequate research on osmolarity, we conduct this study to address these uncertainties. This approach was undertaken because of the relatively low number of patients in many previous investigations and the inconsistencies in their results.

We hypothesized that a comprehensive evaluation of complete blood count (CBC)-associated biomarkers in combination with osmolarity will yield valuable insights into the diagnostic value of these parameters in classifying FSs. Given the limitations of previous studies due to small sample sizes and inconsistent outcomes, we anticipated that CBC biomarkers, along with osmolarity, would provide a more holistic perspective for distinguishing between simple and complex FSs. These laboratory variables reflect aspects of inflammation, immune response, platelet activity, electrolyte balance, and osmolarity, all of which could have implications for the pathophysiology of FSs. Routine blood tests usually have a fast turnaround time, allowing clinicians to obtain results promptly. This rapid availability of information can be crucial in making timely clinical decisions, especially in emergency scenarios.

## 2. Materials and Methods

### 2.1. Ethical Approval

This study followed the principles outlined in the Declaration of Helsinki and obtained approval from both the Ministry of Health of Turkey and the Ethics Committee of Zeynep Kamil Maternity and Children’s Disease Training and Research Hospital. The approval number for this study was 99, and it was granted on 21 June 2023. The use of hospital records was specified in the informed consent forms obtained from parents and legal guardians for diagnosis and treatment during emergency follow-up.

### 2.2. Study Population

We conducted a single-center retrospective study in the pediatric emergency department of the Zeynep Kamil Maternity and Children’s Disease Training and Research Hospital, University of Health Sciences, Istanbul, Turkey, a tertiary-level hospital. We collected data from 755 patients aged 5–60 months (the typical age of febrile seizures) [1,2] admitted to the emergency department due to seizures with fever between 1 January 2020 and 1 June 2023. This study included 364 children with FSs. Fever-associated seizure (FS) is characterized as a seizure occurring in infants and children aged 6 to 60 months, which is accompanied by fever with a body temperature of 38.0 °C or higher, and does not result from a central nervous system infection [5]. We excluded patients with known epilepsy, central nervous system problems, metabolic issues, a history of afebrile seizures, prior indications of an intracranial infection or developmental anomaly, chromosomal abnormalities, a history of intracranial surgery, children with gastroenteritis, and children with missing data.

Finally, 364 children with FSs were included in this study. None of the patients meeting the inclusion criteria exhibited the acute symptomatic conditions known to trigger childhood seizures, such as bacterial meningitis, acute encephalitis, or encephalopathy. The International Classification of Diseases, 10th Revision (ICD-10) codes (ICD-9 780.31, 780.32), was used to diagnose FSs, and the medical reports were examined.

**Figure 1:**
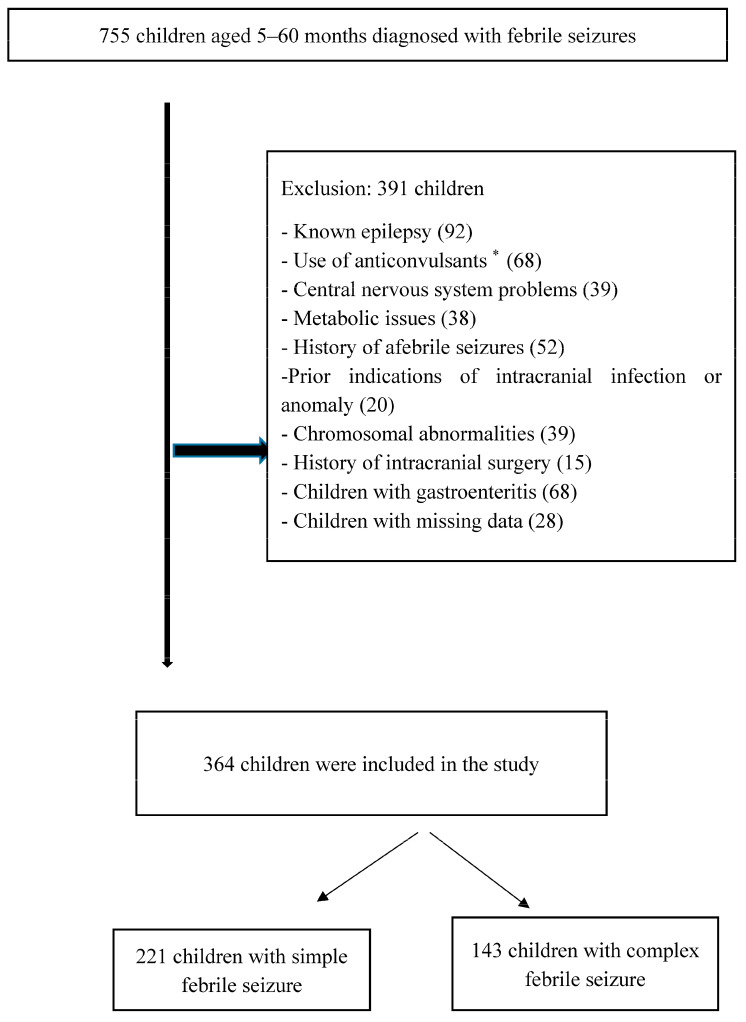
Flowchart of the study enrollment. * Anticonvulsant use was found in different groups.

Diagnostic information for each patient, including seizure classification, medical history, and number of previous FSs, was retrieved from electronic medical records. Two patient groups were established: the first included 221 patients with simple FSs and the second included 143 patients with complex FSs (Figure 1).

A simple FS was defined as a singular seizure lasting < 10 min, occurring no more than once within 24 h, and lacking any focal features. A complex FS was identified by seizures exceeding 10 min (prolonged seizure), occurring more than once in a 24 h timeframe (multiple seizures), or displaying focal features or postictal paresis [5,19]. The number of previous FS episodes, family history of FS, and diagnosis of underlying fever were recorded. Two independent medical professionals reviewed the data and confirmed the consistency of the diagnostic classification. Any discrepancies were resolved by consensus.

Venous blood samples were collected within 30 min of admission and placed in ethylenediaminetetraacetic acid (EDTA)-containing anticoagulant tubes. CBC analysis, including white blood cell (WBC), neutrophils, lymphocytes, platelet count (PLT), MPV, and hemoglobin (Hg) levels, and biochemical tests, including sodium (Na^+^), potassium (K^+^), chloride (Cl^−^), glucose, blood urea nitrogen (BUN), and C-reactive protein (CRP) levels, were performed. The NLR, MPR, and osmolarity were calculated. Osmotic pressure was calculated using the following formula: osmotic pressure (mOsm/kg H2 O) = Na (mEq/L) × 2 + glucose (mg/dL)/18 + BUN (mg/dL)/2.8 [20]. NLR was calculated by dividing NLR and MPR was calculated by dividing MPV by the PLT.

### 2.3. Statistical Analyses

Statistical analyses were conducted using the Number Cruncher Statistical System (NCSS 2007 Statistical Software version 1 (Kaysville, UT, USA). When evaluating the data, in addition to descriptive statistical methods (mean and standard deviation), the distribution of variables was assessed for normality utilizing the Shapiro–Wilk normality test and also the Kolmogorov–Smirnov test. To compare normally distributed variables within paired groups, the independent t-test was employed. For non-normally distributed variables within paired groups, the Mann–Whitney U test was applied. Qualitative data were compared using the chi-squared test. Logistic regression analysis was conducted to identify influential factors. To identify the significant variables for the differential diagnosis, an analysis was conducted employing the receiver operating characteristic (ROC) curve, with a targeted area under the ROC curve threshold set at 0.7 [21]. Sensitivity, specificity, positive predictive value, negative predictive value, and likelihood ratio (LR [+]) were computed to establish the cutoff point. Bonferroni correction was used in multiple comparisons in our study. The findings were assessed at a significance threshold of *p* < 0.05. The considered sample size demonstrated the sufficiency of our statistics due to the larger sample size compared with six previously conducted retrospective cross-sectional studies [15,18,22,23,24,25].

## 3. Results

During the study period, 755 patients were admitted to the pediatric emergency department with FSs. After excluding patients who were unsuitable for the study and those whose laboratory data were missing, 364 patients were included.

Of the 364 patients, 221 (60.72%) had simple FSs and 143 (39.28%) had complex FSs, including 62 with multiple seizures during the same febrile illness and 81 with prolonged seizures; in contrast, upper respiratory infections were the most diagnosed disease in both groups. The baseline characteristics and the laboratory test results of patients are shown in Table 1.

### 3.1. Results of the Simple and Complex FS Groups

The distribution of a family history of febrile seizures within the complex febrile seizure group was notably higher compared to that within the simple febrile seizure group (*p* = 0.009). Neutrophil averages in the complex FS group were significantly higher than those in the simple FS group (*p* = 0.028). The average osmolarity in the complex FS group was significantly lower than that in the simple FS group (*p* = 0.0001). The averages of Na^+^ and Cl^−^ in the complex FS group were significantly lower than those in the simple FS group (*p* = 0.0001 and *p* = 0.002, respectively). However, no other laboratory variables varied significantly according to the seizure type. Logistic regression analysis was performed with family history, neutrophil count, osmolarity, and Na^+^ and glucose variables to determine the factors affecting the presence of complex FSs. Neutrophil count (*p* = 0.114), osmolarity (*p* = 0.493), and glucose (*p* = 0.780) levels were statistically insignificant. In contrast, a high frequency of family history (*p* = 0.004) and low Na^+^ levels (*p* = 0.001) were identified as significant influencing factors. 

**Figure 2:**
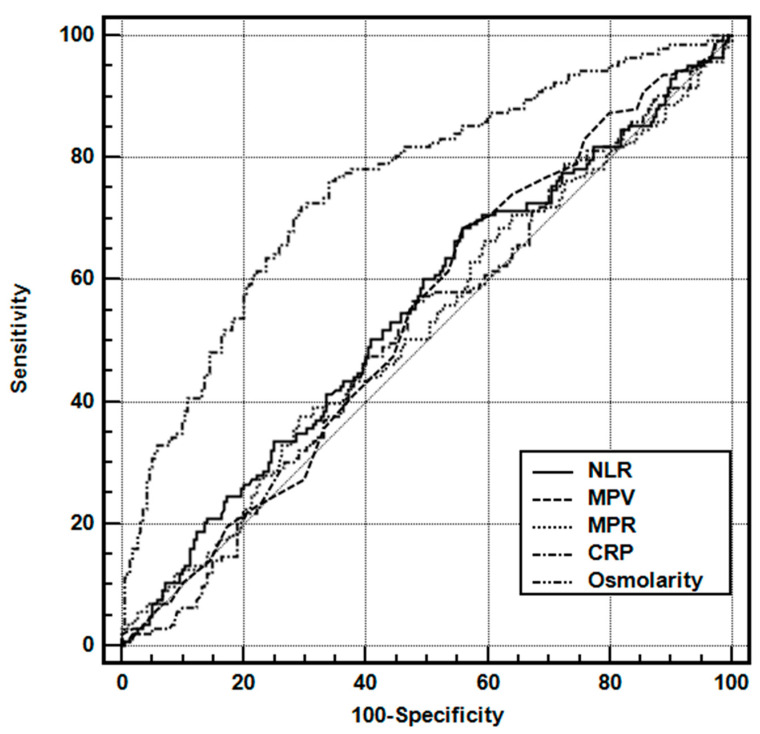
Receiver operating characteristic curves of the various parameters for predicting complex FSs on admission. Abbreviations: NLR, neutrophil-to-lymphocyte ratio; MPV, mean platelet volume; NLR, neutrophil-to-lymphocyte ratio; MPR, MPV-to-platelet ratio.

ROC curve analysis was performed to assess the predictive values of NLR, MPR, MPV, CRP, and osmolarity. In the differential diagnosis of complex FSs, the area under the osmolarity ROC curve was 0.754 (0.707–0.798). In contrast, no other variability was found below the desired limit of 0.700 (Table 2, Figure 2).

For a cutoff of ≤280.04, osmolarity had a sensitivity of 72.03%, specificity of 70.14%, positive predictive value of 60.9%, negative predictive value of 79.5%, and an LR (+) value of 2.41.

### 3.2. Results of Single and Multiple Seizures for Simple and Complex FS Groups

In the simple FS group, the mean age in the ≥ 2 seizure group was significantly higher than that in the single-seizure group (*p* = 0.0001). The distribution of family history in the ≥2 seizure group was significantly higher than that in the single-seizure group (*p* = 0.0001) (Table 3).

**Figure 3:**
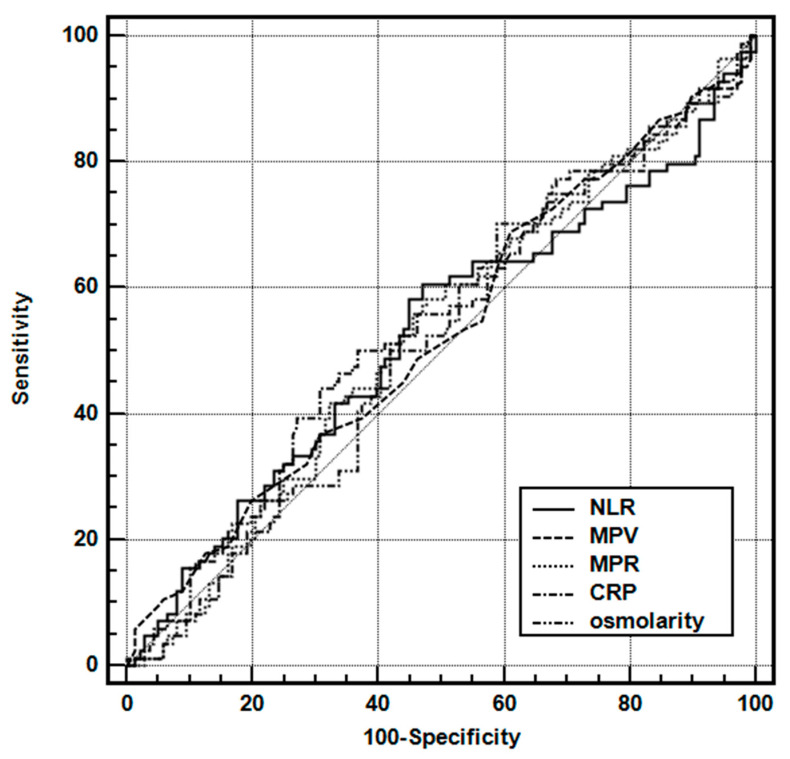
Receiver operating characteristic curves of the various parameters for predicting multiple seizures in the simple FS group. Abbreviations: NLR, neutrophil-to-lymphocyte ratio; MPV, mean platelet volume; NLR, neutrophil-to-lymphocyte ratio; MPR, MPV-to-platelet ratio.

The area under the ROC curve for all laboratory variables was below the desired limit of 0.700 (Table 4, Figure 3).

In the complex FS group, the distribution of family history in the ≥2 seizure group was significantly higher than that in the single-seizure group (*p* = 0.014). The mean Na^+^ level of the ≥ 2 seizure group was significantly higher than that of the single-seizure group (*p* = 0.04). The mean seizure duration in the ≥ 2 seizure group was significantly lower than that in the single-seizure group (*p* = 0.002) (Table 5).

**Figure 4:**
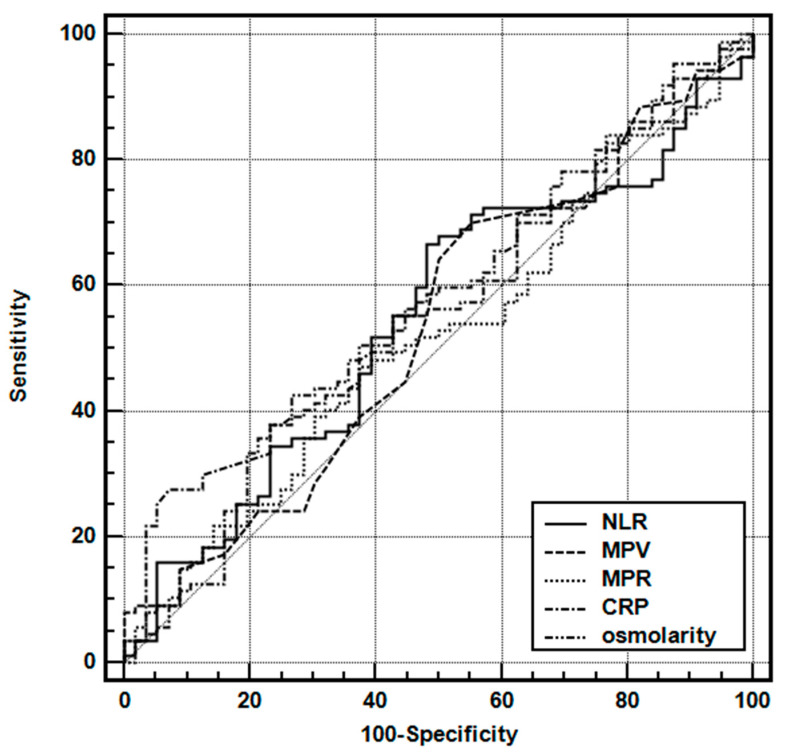
Receiver operating characteristic curves of the various parameters for predicting multiple seizures in the complex FS group. Abbreviations: NLR, neutrophil-to-lymphocyte ratio; MPV, mean platelet volume; NLR, neutrophil-to-lymphocyte ratio; MPR, MPV-to-platelet ratio.

The area under the ROC curve for all laboratory variables was below the desired limit of 0.700 (Table 6) (Figure 4).

## 4. Discussion

The accurate identification of seizure type may be important in developing an appropriate clinical strategy for patients with FSs [26,27]. This study showed that the osmolarity averages in the complex FS group were significantly lower than those in the simple FS group, and the evaluated biomarkers, including NLR, MPV, MLR, and CRP, did not differ significantly between the simple and complex FS groups. Family history and low Na^+^ levels were determined to be significant factors. FSs are categorized into two main types: simple and complex. Simple FSs make up the majority FS cases, accounting for approximately 70–75% of all cases. Our study’s ratio of simple FS to complex FS cases (60.72%) may be slightly lower. This could be because our hospital is a tertiary care facility, resulting in more patients with complex FSs seeking treatment at our institution. In a study conducted by Yazar et al. [28] among patients with simple FSs, acute gastroenteritis was the most frequently identified disease (33.3%).

Conversely, acute tonsillopharyngitis took the lead regarding diagnosis among patients with complex FSs (34%). In our study, upper respiratory tract infections were the most commonly diagnosed diseases, and we excluded patients with acute gastroenteritis because of osmolarity calculation. FS incidence was higher in boys, and the mean age of our study population was also similar to that in previous studies [15,17,23]. Numerous studies, including descriptive human investigations and experimental research, have consistently demonstrated elevated levels of pro-inflammatory cytokines, such as IL-1β, IL-6, and tumor necrosis factor alpha, in individuals affected by FSs [29,30].

While cytokines are commonly used as indicators of inflammation in investigating FSs, a significant issue associated with these markers is their limited accessibility and high costs.

NLR is a dependable, easily measurable, cost-effective, and accessible marker of inflammation. This reflects the equilibrium between two vital blood constituents: lymphocytes, which are protective and regulatory agents, and neutrophils, which are crucial inflammatory elements that release proinflammatory products. Limited reports have been published regarding the functions of these cells [29,31].

Romanowska et al. [32] demonstrated a significant increase in neutrophil levels among children with febrile seizures in comparison to those experiencing fever without seizures. Additionally, children with febrile seizures exhibited a lower count of lymphocytes compared to those without seizures accompanying their fever. In our study, although neutrophil counts were normal, the mean neutrophil levels within the complex febrile seizure group were notably greater than those observed in the simple febrile seizure group, possibly because neutrophil counts can swiftly surge in response to vigorous skeletal muscle action (such as seizures or chills). This surge may stem from an inflammatory response that becomes evident after approximately 4–5 h. Alternatively, it may also be linked to the circulation of harmful toxins in the bloodstream [33]. In addition, the increase in the NLR ratio due to neutrophilia and lymphopenia, which are associated with elevated cortisol levels, was not surprising [17]. In our study, we found that the lymphocyte levels were normal. No significant differences were observed between the simple and complex FS groups. The absence of lymphopenia may be associated with diverse inflammatory responses, individual variations, lymphocyte distribution frequencies, and seizure integrity. Given the distinct management strategies and approaches necessary for each type of FS, it is essential to classify them. However, it is important to note that these conclusions may not be universally applicable. For most FS cases, differentiation between simple and complex FSs can be effectively achieved through physical examination and assessing comprehensive medical history obtained from the parents [1]. Therefore, a CBC test and precise NLR measurements may not be necessary for many patients with FSs. Distinguishing between simple and complex FSs can be perplexing due to the altered clinical indicators of illness upon admission. This complexity can arise from factors such as the administration of anticonvulsive therapy during the patient’s transfer to the hospital and challenges in obtaining a comprehensive medical history from parents who may be in an agitated state [1]. Therefore, without a reliable medical history or physical examination, clinical research has sought to investigate the association between MPV, NLR, PLT, and red-cell distribution (RDW) levels in the blood of febrile children with and without seizures; however, these investigations have produced conflicting findings. In recent years, NLR has been proposed as a practical predictor of FS-type differentiation [15,23,24,25,34]. However, the sample size of these studies was smaller than that of our study.

In a meta-analysis by Hosseini et al. involving 11 studies associated with NLR to distinguish simple and complex FSs, NLR levels in patients with complex febrile seizures were notably elevated compared to those in the simple febrile seizure group. Consequently, one could reasonably hypothesize that the degree of inflammation in patients with complex febrile seizures is greater than that in patients with simple febrile seizures, suggesting that inflammation potentially has a more prominent role in the development of complex febrile seizures compared to simple ones. However, the studies were categorized into three subgroups based on sample sizes: small (with sample sizes ≤ 100), medium (with sample sizes between 100 and 200), and large (with sample sizes exceeding 200) [16]. Our study included 364 patients. In this meta-analysis, studies involving limited and medium sample sizes showed that NLR in patients with complex FSs exhibited a statistically significant increase compared to the NLR observed in cases of simple FSs. However, the NLR did not differ in simple and complex FS cases in large studies. Based on this categorization, our study included a large sample size of 364 patients. A significant limitation of that meta-analysis is the limited sample size of the studies included in the analysis. Consequently, the potential impact of the findings may be diminished, and further studies should be conducted to enhance the robustness of their results. Moreover, the studies included in our analysis exhibited considerable heterogeneity.

Liu et al. demonstrated that NLR serves as an independent predictor, although with some limitations, in distinguishing between simple and complex febrile seizures. Furthermore, there is a suggestion that NLR and MPR may exhibit synergistic effects that can impact the occurrence of febrile seizures [17]. Kubato et al. found no relationship between NLR and FS type, regardless of the median NLR value [18]. Tang and Chen identified a strong correlation between PLT (platelet count) and MPV (mean platelet volume) and the onset of febrile seizures. Additionally, they proposed that PLT and platelet distribution width (PDW) could function as straightforward yet effective laboratory markers for distinguishing the various types of febrile seizures. In this study, WBC count, neutrophil ratio, NLR, PLR, and CRP exhibited significantly higher levels in both the febrile seizure (FS) group and the fever group without seizures when compared to the healthy group. Notably, there were no statistically significant differences in these parameters between the FS and fever groups [35].

Similarly, both Romanowska and Liu et al. reported no significant distinctions in white blood cell (WBC) counts when comparing the febrile seizure group to the fever without seizure group [17,29]. Yazar et al. showed that the WBC count and NLR were significantly increased in patients with complex FSs compared to those with simple FSs. MPV values were also increased in patients with complex FSs, which were not statistically significant [28]. However, their sample size was small. A study conducted by Ozaydin et al. compared children with simple and complex FSs. Their findings diverged from our study’s results as they observed a notable reduction in MPV levels among patients with complex FSs [36]. This variance stands in contrast to our study’s outcomes. In classifying febrile convulsions based on hematological parameters, we could not find a significant data point. Although various factors may contribute to febrile convulsions, hematological parameters alone may not serve as reliable indicators for classification. In medical practice, FSs are generally diagnosed and managed based on a comprehensive evaluation of the child’s medical history, physical examination, and additional diagnostic tests if necessary. Hematological parameters may be part of the overall assessment; however, they are not considered primary or significant determinants for classifying febrile convulsions. Acute febrile illness usually results in minor water and electrolyte balance disturbances. Changes in the electrolyte balance, especially hyponatremia, may predispose children to convulsions during febrile illnesses. Andrew found that a lower osmotic pressure contributed to the onset of seizures [37]. Studies on the relationship between osmolarity and FS type are limited. Our study found that, although the median osmotic pressures of all seizure types were below the normal range, the osmolarity averages in the complex FS group were significantly lower than those in the simple FS group. We measured osmolarity by the calculating formula: Na (mEq/L) × 2 + glucose (mg/dL)/18 + BUN (mg/dL)/2.8; however, no influencing factors (such as the administration of mannitol or ethanol) could lead to a disparity between the calculated and actual osmotic pressures. Kubato et al. discovered a significant elevation in osmotic pressure among individuals experiencing the prolonged fever subtype of complex febrile seizures [18].

Our study had several limitations. The primary limitation was its retrospective single-center design, which focused exclusively on a particular patient population. Another limitation was the absence of a control group comprising patients with fever but no seizure. Additionally, we did not include patients with focal seizures, which could potentially introduce a bias in the representation of seizure types. This limitation may affect the generalizability of our findings to a broader population, including patients with focal seizures. Furthermore, our study did not include data on cytokine levels, which may have played a crucial role in understanding the underlying inflammatory mechanisms associated with seizures. The lack of cytokine data limited our ability to fully explore the potential link between inflammation and seizure activity, and the time between seizure onset and blood sampling was not included in our records.

The strength of our study was that it is more reflective of real-world clinical settings than earlier research. This was achieved by incorporating a broader range of participants, including children with a history of FSs and those younger than 1 year. Additionally, the sample size in this study exceeded the sample sizes of prior studies.

Furthermore, the larger sample size improved the statistical robustness and generalizability of the study’s findings compared to earlier, potentially more limited, research efforts.

In conclusion, clinical signs and symptoms were used to categorize FSs. Easily measurable inflammatory markers, including NLR, MPV, MPR, and CRP, could not distinguish between the various types of FSs in children. Children who experienced complex febrile seizures exhibited notably lower osmolarity levels compared to those who had simple febrile seizures.

This study addressed the limitations of established biomarkers in providing definitive differentiation or their limited impact in accurately diagnosing FSs. Our findings demonstrate that inflammatory markers, such as NLR, are ineffective in predicting FS types. However, we observed that cases of complex FSs exhibited significantly lower osmolarity than simple FS cases.

These findings underscore that predetermined biomarkers do not always yield precise results, particularly when diagnosing FSs. Future research directions in this field should explore the combined use of clinical evaluations and biomarkers, aiming for a more effective diagnostic approach for FS cases. A comprehensive exploration of various variables, such as clinical characteristics, risk factors, and seizure frequency, should form a significant roadmap for future investigations in this area. Moreover, the potential clinical applications of osmolarity should be acknowledged. While osmolarity is not commonly assessed in routine clinical practice, its inclusion in studies such as this one aims to explore whether it can offer additional diagnostic value. If osmolarity is found to correlate with the occurrence or severity of febrile seizures, it could become a useful biomarker for clinical assessment. Osmolarity could be considered an additional tool to assist in clinical decision-making. Physicians may consider incorporating osmolarity as an evaluative tool to support clinical judgment and enhance diagnostic accuracy.

## Figures and Tables

**Table 1:** Independent sample tests. † Mann–Whitney U test. + Chi-squared test.

		Total Patients *n* = 364		Patients with Simple FSs *n* = 221		Patients with Complex FSs *n* = 143		*p*
**Age (months)**		28.34 ± 15.58		28.51 ± 15.97		28.09 ± 15.01		0.804 *
**Sex**	**Male**	151	65.66%	151	68.33%	88	61.54%	0.183 +
	**Female**	125	34.34%	70	31.67%	55	38.46%	
**Family history FS**	**Present**	199	54 > 67%	133	60.18%	66	46.15%	**0.009** +
	**Absent**	165	45.33%	88	39.82%	77	53.85%	
**Diagnosis**	**Bronchiolitis**	5	1.37%	4	1.81%	1	0.70%	0.287 +
	**Urinary tract infection**	10	2.75%	8	3.62%	2	1.40%	
	**Otitis media**	48	13.19%	34	15.38%	14	9.79%	
	**Pneumonia**	9	2.47%	4	1.81%	5	3.50%	
	**Tonsillitis**	17	4.67%	9	4.07%	8	5.59%	
	**URTI**	275	75.55%	162	73.30%	113	79.02%	
**Seizure**	**Once in 24 h**	302	82.97%	221	100.00%	81	56.64%	**0.0001** +
	**Twice in 24 h**	62	17.03%	0	0.00%	62	43.36%	
**Duration of fever (days)**		1.43 ± 0.69		1.45 ± 0.74		1.4 ± 0.62		0.471 †
**Duration of seizure (min)**		6.07 ± 7.74		2.42 ± 2.2		11.7 ± 9.63		**0.0001** †
**Number of previous seizure**		2.03 ± 1.61		1.94 ± 1.74		2.17 ± 1.38		0.190 †
**WBC count (10^9^/L)**		12.23 ± 5.86		11.75 ± 5.35		12.97 ± 6.51		0.053 *

**Neutrophil (10^9^/L)**		7.95 ± 5.06		7.48 ± 4.53		8.68 ± 5.73		**0.028** *

**Lymphocyte (10^9^/L)**		3.21 ± 1.8		3.21 ± 1.76		3.2 ± 1.86		0.974 *
**Hemoglobin (g/dL)**		11.61 ± 1.08		11.66 ± 1.03		11.52 ± 1.15		0.219 *
**Platelet (10^9^/L)**		292.5 ± 98.82		290.85 ± 92.5		295.04 ± 108.11		0.693 *
**MPV (f/L)**		8.1 ± 0.88		8.17 ± 0.8		8 ± 0.98		0.076 *
**NLR**		3.46 ± 3.34		3.32 ± 3.37		3.67 ± 3.28		0.118 †
**MPR**		0.03 ± 0.03		0.03 ± 0.01		0.03 ± 0.05		0.489 †
**CRP (mg/L)**		20.59 ± 31.26		21.68 ± 33.44		18.91 ± 27.59		0.411 †
**Na^+^** (mEq/L)		135.24 ± 2.53		136.13 ± 2.21		133.87 ± 2.39		**0.0001** *
**K^+^** (mEq/L)		4.35 ± 0.43		4.37 ± 0.44		4.34 ± 0.4		0.527 *
**Cl^−^** (mEq/L)		102.05 ± 3.13		102.46 ± 2.92		101.42 ± 3.35		**0.002** *
**Glucose** (mg/dL)		111.46 ± 25.86		110.46 ± 23.83		113.01 ± 28.74		0.360 *
**BUN**		10.98 ± 3.3		11.14 ± 3.44		10.74 ± 3.08		0.255 *
**Osmolarity** (mOsmol/L)		280.59 ± 5.22		282.37 ± 4.63		277.85 ± 4.88		**0.0001** *

* Independent sample test. † Mann–Whitney U test. + Chi-squared test. Bold values indicate statistical differences, and correlations were considered significant at *p* < 0.05. Abbreviations: CRP, C-reactive protein; MPV, mean platelet volume; NLR, neutrophil-to-lymphocyte ratio; WBC, white blood cell; PLR, platelet-to-lymphocyte ratio; MLR, MPV to lymphocyte ratio; URT, upper respiratory tract.

**Table 2:** Receiver operating characteristic curve analysis of the laboratory parameters for predicting complex FSs on admission.

	AUC	SE	95% CI
NLR	0.549	0.031	0.496–0.600
MPV	0.540	0.031	0.487–0.592
MPR	0.521	0.031	0.469–0.574
CRP (mg/L)	0.516	0.031	0.464–0.569
Osmolarity (mOsmol/L)	0.754	0.025	0.707–0.798

Abbreviations: NLR, neutrophil-to-lymphocyte ratio; MPV, mean platelet volume; MPR, MPV-to-platelet ratio; CRP, C-reactive protein; AUC, area under the curve; CI, confidence interval.

**Table 3:** Baseline characteristics and laboratory test results of patients with simple FSs with single and multiple seizures.

Simple FSs		Single Seizure, *n* = 136		≥2 Seizures, *n* = 136		*p*
**Age (months)**		23.65 ± 14.05		36.27 ± 15.86		**0.0001** *
**Sex**	**Male**	96	70.59%	55	64.71%	0.360 +
	**Female**	40	29.41%	30	35.29%	
**Family History**	**Present**	102	75.00%	31	36.47%	**0.0001** +
	**Absent**	34	25.00%	54	63.53%	
**Duration of fever (days)**		1.48 ± 0.78		1.41 ± 0.68		0.519 †
**Duration of seizure (min)**		2.51 ± 2.26		2.27 ± 2.1		0.424 †
**WBC (10^9^/L)**		11.56 ± 5.76		12.06 ± 4.65		0.508 *
**Neutrophil (10^9^/L)**		7.41 ± 4.79		7.6 ± 4.1		0.770 *
**Lymphocyte (10^9^/L)**		3.14 ± 1.7		3.32 ± 1.85		0.473 *
**Hemoglobin (g/dL)**		11.64 ± 1.01		11.69 ± 1.06		0.730 *
**Platelet (10^9^/L)**		290.85 ± 97.04		290.86 ± 85.2		0.999 *
**MPV (f/L)**		8.19 ± 0.77		8.13 ± 0.85		0.589 *
**NLR**		3.22 ± 3.43		3.47 ± 3.3		0.566 †
**MPR**		0.03 ± 0.02		0.03 ± 0.01		0.517 †
**CRP (mg/L)**		21.16 ± 33.28		22.49 ± 33.88		0.775 †
**Na^+^** (mEq/L)		136.06 ± 2.27		136.24 ± 2.11		0.564 *
**K^+^** (mEq/L)		4.34 ± 0.43		4.4 ± 0.45		0.334 *
**Cl^−^** (mEq/L)		102.67 ± 3.08		102.14 ± 2.63		0.195 *
**Glucose** (mg/dL)		112.13 ± 23.77		107.8 ± 23.82		0.189 *
**BUN** (mg/dL)		11.25 ± 3.57		10.96 ± 3.24		0.553 *
**Osmolarity** (mOsmol/L)		282.36 ± 4.66		282.38 ± 4.61		0.986 *

* Independent sample test. † Mann–Whitney U test. + Chi-squared test. Bold values indicate statistical differences, and correlations were considered significant at *p* < 0.05. Abbreviations: CRP, C-reactive protein; MPV, mean platelet volume; NLR, neutrophil-to-lymphocyte ratio; WBC, white blood cell; PLR, platelet-to-lymphocyte ratio; MLR, MPV-to-lymphocyte ratio.

**Table 4:** Receiver operating characteristic curve analysis of the laboratory parameters for predicting multiple FSs in the simple FS group.

	AUC	SE	95% CI
NLR	0.523	0.0402	0.455–0.591
MPV	0.527	0.0399	0.459–0.594
MPR	0.526	0.0399	0.458–0.594
CRP (mg/L)	0.534	0.0403	0.466–0601
Osmolarity (mOsmol/L)	0.518	0.0402	0.450–0.586

Abbreviations: CRP, C-reactive protein; MPV, mean platelet volume; NLR, neutrophil-to-lymphocyte ratio; MPR, MPV-to-platelet ratio.

**Table 5:** Baseline characteristics and laboratory test results of patients with complex FSs with single and multiple seizures.

Complex FSs		Single Seizure, *n* = 56		≥2 Seizures, *n* = 65		*p*
**Age (months)**		27.21 ± 14.55		28.66 ± 15.36		0.577 *
**Sex**	**Male**	33	58.93%	55	63.22%	0.607 +
	**Female**	23	41.07%	32	36.78%	
**Family history**	**Absent**	33	58.93%	33	37.93%	**0.014** +
	**Present**	23	41.07%	54	62.07%	
**Duration of fever**		1.32 ± 0.58		1.45 ± 0.64		0.232 *
**Duration of seizure**		14.8 ± 8.83		9.7 ± 9.65		**0.002** †
**WBC (10^9^/L)**		12.63 ± 6.66		13.19 ± 6.44		0.618 *
**Neutrophil (10^9^/L)**		8.54 ± 5.64		8.76 ± 5.81		0.824 †
**Lymphocyte (10^9^/L)**		3.01 ± 1.97		3.33 ± 1.79		0.322 *
**Hemoglobin (g/dL)**		11.65 ± 1.07		11.44 ± 1.2		0.279 *
**Platelet (10^9^/L)**		300.5 ± 115.4		291.53 ± 103.68		0.630 *
**MPV (fL)**		7.99 ± 0.65		8.01 ± 1.14		0.878 *
**NLR**		3.65 ± 2.74		3.69 ± 3.61		0.346 †
**MPR**		0.04 ± 0.07		0.03 ± 0.02		0.702 †
**CRP (mg/L)**		20.79 ± 27.58		17.7 ± 27.69		0.516 †
**Na^+^** (mEq/L)		133.36 ± 2.31		134.2 ± 2.4		**0.04** *
**K^+^** (mEq/L)		4.26 ± 0.43		4.39 ± 0.38		0.076 *
**Cl^−^** (mEq/L)		101.23 ± 2.94		101.55 ± 3.6		0.568 *
**Glucose** (mg/dL)		118.56 ± 34.56		109.43 ± 23.82		0.064 *
**BUN** (mg/dL)		11.01 ± 3.38		10.56 ± 2.87		0.392 *
**Osmolarity** (mOsmol/L)		277.23 ± 4.99		278.24 ± 4.8		0.229 *

* Independent sample test. † Mann–Whitney U test. + Chi-squared test. Bold values indicate statistical differences and correlations were considered significant at *p* < 0.05. Abbreviations: CRP, C-reactive protein; MPV, mean platelet volume; NLR, neutrophil-to-lymphocyte ratio; WBC, white blood cell; PLR, platelet-to-lymphocyte ratio; MLR, MPV-to-lymphocyte ratio.

**Table 6:** Receiver operating characteristic curve analysis of the laboratory parameters for predicting multiple FSs in the complex FS group.

	AUC	SE	95% CI
NLR	0.547	0.050	0.461–0.630
MPV	0.535	0.049	0.450–0.619
MPR	0.519	0.050	0.434–0.603
CRP (mg/L)	0.575	0.050	0.489–0.657
Osmolarity (mOsmol/L)	0.557	0.049	0.472–0.640

Abbreviations: CRP, C-reactive protein; MPV, mean platelet volume; NLR, neutrophil-to-lymphocyte ratio; MPR, MPV-to-platelet ratio.

## Data Availability

The datasets generated during and/or analyzed during the present study are available from the corresponding author upon reasonable request.
